# Recent advances in managing systemic sclerosis

**DOI:** 10.12688/f1000research.10022.1

**Published:** 2017-01-30

**Authors:** Martin Aringer, Anne Erler

**Affiliations:** 1 Division of Rheumatology, Medicine III, University Medical Center and Faculty of Medicine Carl Gustav Carus at the TU Dresden, Dresden, Germany

**Keywords:** systemic sclerosis, scleroderma, SSc, ACE inhibitors, phosphodiesterase 5 blockers

## Abstract

How the main components in systemic sclerosis—namely autoimmunity, vasculopathy, and fibrosis—fit together is still not sufficiently clear. However, vascular treatment options are well established, the body of evidence for the efficacy of immunomodulatory approaches is increasing, and now at least one hopeful substance that may directly interfere with fibrosis is being tested. Although we still wait for important breakthroughs, there is grounds for hope that better therapeutic options will be available in the near future.

Most rheumatologists would agree that systemic sclerosis (SSc) still can be a dreadful disease and that the advances we see in other areas have not yet arrived. Therapies for SSc found effective in randomized controlled trials (RCTs) are still sparse, and the effect size of these drugs was often quite limited. Nevertheless, significantly advanced by Marco Matucci-Cerinic’s founding of EUSTAR, the European League Against Rheumatism (EULAR) Scleroderma Trials And Research group ^1^, a huge effort has been under way for more than ten years now. Indeed, the last few years have started to change at least the outlook. We still have to wait for the real breakthroughs, but there is hope.

In part, limited progress is caused by not comprehensively understanding the disease ^2, 3^. SSc always has a vascular aspect, resulting in a vasculopathy that is clearly distinguishable from vasculitis and that, at least in the beginning, is immunologically mediated. One of the consequences is (late-onset) Raynaud’s syndrome ^4^, which commonly is an early sign of SSc. Vasculopathy underlies pulmonary arterial hypertension (PAH) and scleroderma renal crisis. Via hypoxia and cytokines such as transforming growth factor beta ^5^, vasculopathy also is one reason for the fibrotic changes in the disease. In diffuse cutaneous SSc (dcSSc), fibrosis is also induced by direct immune system effects on fibroblasts ^6^, and clear inflammatory changes are found in SSc skin and lungs. How these three fit together is not yet sufficiently clear.

However, it has become much more obvious that the growing group of SSc-specific autoantibodies tested in clinical routine are associated with distinct clinical manifestations. In the 2013 American College of Rheumatology/EULAR classification criteria ^7^, it is the autoantibodies against centromeres, topoisomerase I, and RNA polymerase III that the system relies on in addition to vascular changes and puffy fingers or sclerodactyly. These leading autoantibodies also predict SSc classification ^8^ and differentiate typical clinical pictures that include organ manifestation and prognosis ^9, 10^. Unfortunately, they do not usually disappear under current therapeutic approaches, including autologous stem cell transplantation ^11^, suggesting that SSc treatment is suboptimal even in the most drastic regimens used today. Another aspect, which has been brought forward, is that at least anti-RNA polymerase III antibodies may also herald paraneoplastic SSc, and older age at onset, mostly more than 50 years, and less pronounced Raynaud’s also weigh in 12. There is accumulating, albeit still circumstantial, evidence that these autoantibodies cannot be reduced to a bystander phenomenon, even if a direct pathogenetic role has yet to be defined.

In addition, autoantibodies against endothelial receptors have been found in the sera of many patients with SSc and are associated with worse outcome ^13, 14^. This is well in line with the major vascular symptoms of patients with SSc, ranging from severe Raynaud’s to PAH and renal crisis. These antibodies are not part of the routine work-up today, and the extent of their influence and the influence of potential other autoantibodies that target endothelial cells will have to be determined. Nevertheless, the story is intriguing.

On the probable effector side of these antibodies, nailfold capillary microscopy is now a well-established tool to evaluate capillary damage ^15^. Indeed, changes over time have been demonstrated. The presence of later stages of capillary damage is a predictor of severe vascular complications, such as SSc digital ulcers. However, there is still need for reliable microvascular outcome parameters, which would allow early therapeutic effects to be differentiated. Capillary microscopy is helpful in recognizing early disease ^4^. However, in a small cohort study, megacapillaries in the absence of SSc-specific autoantibodies were not associated with fulfilling SSc classification criteria in the near future ^8^.

Today’s established therapeutic approaches that are based on clinical facts work either on the vascular side or on the inflammatory side. Anti-fibrotic drugs have not yet been shown to work for SSc if one does not see methotrexate as a partially anti-fibrotic agent. However, at least one putatively anti-fibrotic substance, nintedanib, is being tested for SSc interstitial lung disease (see below).

On the vascular side, angiotensin-converting enzyme inhibitors have greatly improved the outcome of SSc renal crisis. However, this still constitutes a dramatic situation with a high rate of death and renal failure ^16, 17^. Two RCTs showed bosentan to be effective for preventing SSc digital ulcers but failed to show effects in healing ^18^. This endothelin receptor antagonist (ERA) treats PAH also in patients with SSc, prolonging survival, as do other ERAs not tested for digital ulcers ^19^. However, macitentan, which is approved for PAH, failed to show efficacy for SSc digital ulcers ^20^.

Limited evidence suggests that iloprost may improve ulcer healing ^18^. In addition, there is increasing evidence of a positive effect of phosphodiesterase 5 (PDE5) blockers, such as sildenafil and tadalafil ^18^. Tadalafil had been found to be effective for both ulcer healing and prophylaxis in a small controlled cross-over trial ^21^. Sildenafil failed to meet its primary endpoint in the French SEDUCE trial; time to healing showed only a trend ( *P* = 0.18) toward sildenafil benefit. Significant results in secondary analyses of these data, such as the number of ulcers at 8 ( *P* = 0.01) and 12 ( *P* = 0.03) weeks, still suggest a real influence ^22^, which may take slightly more time than expected.

These drugs also work in PAH, including SSc PAH, alone and in combinations ^19^. Their efficacy in PAH has been known for several years, and only the oral selective prostacyclin receptor agonist selexipag constitutes a novel approach ^23^. However, three of the novel large PAH trials—namely GRIPHON ^23^, SERAPHIN ^24^, and AMBITION ^25^—have clearly shown the value of oral PAH combination therapy, with selexipag plus PDE5 blockers or ERAs or both, the ERA macitentan plus PDE5 inhibitors, and the ERA ambrisentan plus the PDE5 inhibitor tadalafil, respectively. The last of these were also used in early combination in a successful open-label trial in SSc-associated PAH ^26^.

On the inflammatory side, ten years ago, the Scleroderma Lung Study had shown that cyclophosphamide had a limited but significant effect on the deterioration of SSc interstitial lung disease ^27^. As with other diseases, such as systemic lupus erythematosus (SLE) ^28^ or anti-neutrophil cytoplasmic antibody (ANCA)-associated vasculitides ^29^, stopping immunosuppression after cyclophosphamide apparently is not a successful concept. In fact, after cyclophosphamide in the treatment arm was stopped, the differences between the two arms were lost rapidly ^30^. At least, cohort data now suggest that the effect of a cyclophosphamide regimen on SSc interstitial lung disease can be stabilized by azathioprine ^31^. With a somewhat unusual intravenous cyclophosphamide protocol, in which a total dose of 10 g was administered in weekly 500 mg infusions, 23% had improved and an additional 38% had stable forced vital capacity (FVC) with stable diffusion capacity of the lung for carbon monoxide (DLCO). Of these 24 patients, all but three (13%) remained at least stable under azathioprine (2 mg/kg every day). In a comparable approach, 20 French patients received 12 g cyclophosphamide (in monthly bolus infusions) followed by mycophenolate mofetil (MMF). After cyclophosphamide, 35% had improved and an additional 50% stabilized under cyclophosphamide, but lung function declined in a few additional patients under MMF, resulting in 70% improved or stable after cyclophosphamide and 6 months of MMF ^32^. These data are also supported by Australian cohort data on 29 patients under azathioprine and 22 under MMF, three quarters of whom had received cyclophosphamide before, suggesting stabilization (or improvement) for the majority of patients ^33^.

In the hope of further improving outcome in early dcSSc by a more aggressive approach, the ASTIS (Autologous Stem Cell Transplantation International Scleroderma) trial has compared autologous stem cell transplantation (ASCT) with cyclophosphamide ^34^. ASTIS indeed found improved longer-term outcomes for ASCT but at the price of considerable (10%) procedure-related early mortality. In contrast to SLE, in which ASCT commonly leads to the disappearance of autoantibodies ^35^, SSc autoantibodies typically persist, as already mentioned above, and Raynaud’s also typically remains a problem.

On the other side, with the idea to reduce the risk for adverse events, the Scleroderma Lung Study 2 compared one year of oral cyclophosphamide with two years of MMF in doses of up to 3 g every day in patients with SSc interstitial lung disease ^36^. As compared with deteriorating lung function in the placebo arm in the Scleroderma Lung Study 1 ^27^, lung function improved both in the cyclophosphamide and the MMF arm in the Scleroderma Lung Study 2. There was no significant difference between the treatment arms, but more patients died and more patients left the study in the cyclophosphamide group. Accordingly, MMF may be an appropriate option for induction therapy.

In case of cyclophosphamide failure, MMF may not be sufficient for stabilizing interstitial lung disease: in the above-mentioned Italian cohort study ^31^, only four of 12 patients refractory to cyclophosphamide at least stabilized and none improved. However, a small Turkish cohort had somewhat more favorable results; the majority of patients with cyclophosphamide-refractory interstitial lung disease at least stabilized under MMF ^37^. For the B cell-depleting anti-CD20 antibody rituximab, there is at least some evidence that it may work for SSc interstitial lung disease if cyclophosphamide has failed ^38^, and an antibody against the CD19 B cell receptor showed indications of efficacy in a phase I trial ^39^. 

Excitingly, two entirely novel approaches are currently being tested in large RCTs. One is interleukin-6 (IL-6) receptor blockade with tocilizumab, for which an n = 87 phase II RCT has been published, and another, larger one is ongoing. IL-6 has been shown to be highly expressed in SSc skin, and tocilizumab showed a trend toward improving both skin and lung involvement. The phase II faSScinate trial has not met its primary endpoint, a difference in improvement in skin thickening as per modified Rodnan’s skin score (mRSS), despite a trend ( *P* = 0.09) in this direction ^40^. From 26 ± 5.9 and 26 ± 7.2 at randomization, mRSS improved to 21.8 ± 9.9 and 23.2 ± 9.3 by week 24 for tocilizumab and placebo, respectively, and to 19.6 ± 10.1 and 22.3 ± 8.1 by week 48. Higher percentages of patients under tocilizumab had stabilization of their interstitial lung disease at 24 weeks ( *P* = 0.009) and 48 weeks ( *P* = 0.037). Provided that the ongoing RCT confirms this improvement, tocilizumab may well become the first SSc biological.

On the other hand, there is an ongoing RCT with a mostly anti-fibrotic approach: Nintedanib, which has shown stabilization and survival benefits for patients with idiopathic lung fibrosis ^41– 43^, is hoped to show similar benefit for SSc interstitial lung disease. Although nintedanib acts downstream of receptors for pro-fibrotic cytokines, including the vascular endothelial growth factor (VEGF), fibroblast growth factor (FGF), and platelet-derived growth factor (PDGF) receptors, this drug could also influence inflammatory cytokine signaling ^44^. If it works, it will be interesting to focus on the molecular pathways leading there.

There is clear progress in the management of SSc. The diagnostic and classification tools are improved. In addition to the evidence on bosentan and iloprost, there is now some evidence that PDE5 inhibitors have beneficial effects on SSc digital ulcers. Cyclophosphamide followed by either azathioprine or MMF is apparently able to stabilize interstitial lung disease. MMF has emerged as an alternative option for induction therapy. Though associated with significant procedure-related mortality, ASCT further improves survival in severe early dcSSc. Two novel approaches—the IL-6 receptor blocker tocilizumab and nintedanib—are in phase III clinical trials, both of which are based on a rather robust rationale, and if all goes well, we may have new drugs for SSc soon.
